# Structural Characteristics–Reactivity Relationships for Catalytic Depolymerization of Lignin into Aromatic Compounds: A Review

**DOI:** 10.3390/ijms24098330

**Published:** 2023-05-05

**Authors:** Xin Wang, Wenbiao Xu, Dan Zhang, Xiangyu Li, Junyou Shi

**Affiliations:** 1 Key Laboratory of Wooden Materials Science and Engineering of Jilin Province, Beihua University, Binjiang East Road, Jilin 132013, China; wangxin19970305@126.com; 2 Key Laboratory of Biomass Materials Science and Technology of Jilin Province, Beihua University, Binjiang East Road, Jilin 132013, China; dzhang10482844461@163.com; 3 Collaborative Innovation Center of Forest Biomass Green Manufacturing of Jilin Province, Beihua University, Binjiang East Road, Jilin 132013, China; lixyv@126.com

**Keywords:** lignin, depolymerization, structure, aromatic compounds, monomer yield

## Abstract

Developing renewable biomass resources is an urgent task to reduce climate change. Lignin, the only renewable aromatic feedstock present in nature, has attracted considerable global interest in its transformation and utilization. However, the complexity of lignin’s structure, uncertain linkages, stability of side chain connection, and inevitable recondensation of reaction fragments make lignin depolymerization into biofuels or platform chemicals a daunting challenge. Therefore, understanding the structural characteristics and reactivity relationships is crucial for achieving high-value utilization of lignin. In this review, we summarize the key achievements in the field of lignin conversion with a focus on the effects of the β-O-4 content, S/G ratio, lignin sources, and an “ideal” lignin—catechyl lignin. We discuss how these characteristics influence the formation of lignin monomer products and provide an outlook on the future direction of lignin depolymerization.

## 1. Introduction

The massive use of fossil fuels has caused the biggest environmental problem, leading to continuous deterioration [1]. Energy generation from fossil fuels releases significant amounts of carbon dioxide and other harmful gases, resulting in environmental impacts such as the greenhouse effect, acid rain, and ozone layer depletion [2,3,4,5]. The depletion of fossil resources and the resulting climate change have accelerated the development of the bio-based economy, which aims to obtain fuels, platform chemicals, and material precursors from renewable biomass feedstock [6]. Clean and sustainable energy has gained widespread usage over the past decade, meeting the increasing energy demand while mitigating greenhouse gas emissions and air pollution [7,8]. It is imperative to explore alternative energy sources, and biomass, abundant and sustainable in nature, is considered a potential alternative to traditional fossil resources. It can be converted not only into energy and fuel but also into high value-added chemicals [9].

Lignocellulose is the most abundant source of hydrocarbons in biomass, consisting of cellulose (40–45%), hemicellulose (25–35%), and lignin (15–30%). Lignin, in particular, is an excellent candidate for renewable feedstocks and is the most abundant renewable aromatic resource on Earth, but its potential is not fully realized due to its low utilization efficiency and mainly being incinerated as energy waste [10,11,12,13,14]. The development of biorefinery technology focusing on lignocellulose fractionation, lignin depolymerization, and upgrading to target chemicals will be crucial in improving the utilization rate of plant fiber raw materials, achieving economic feasibility and environmental sustainability [15,16]. After lignin degradation, fine chemicals such as bio-oil can be prepared. In addition, due to the abundant oxygen-containing active groups (such as phenolic hydroxyl group) in the side chain, lignin can also be combined with other chemicals to prepare lignin-based polymer materials [17]. Lignin-based polymer materials mainly include films [18], adhesives [19], hydrogel [20], resins [21], poly lactic acid [22], etc. The complex structure of lignin, wide range of raw material sources and separation methods, low yield of depolymerization products, poor selectivity, and difficulties in characterization have hindered its effective utilization [23,24]. Although much research has focused on lignin separation and depolymerization, few studies have investigated the relationship between lignin structure and its catalytic depolymerization process [25,26,27]. Therefore, a deeper understanding of lignin structure can contribute to developing a more effective lignin depolymerization mechanism and utilizing lignin in a more valuable way.

The transformation of lignin is influenced by a variety of factors. For instance, the addition of inhibitors, such as supercritical fluids, can not only penetrate the molecular structure of lignin quickly and accelerate its dissolution but also inhibit or reduce the occurrence of lignin intermediate repolymerization reactions. This reduction in reactions leads to a decrease in the production of coke, ultimately improving the degradation rate of lignin [28]. Supercritical ethanol can also act as a capping agent, reacting with lignin fragments through alkylation to enhance the stability of phenolic intermediates [29]. Solvents play a crucial role in lignin liquefaction and depolymerization, and solvent selection also affects product yield and selectivity. The functions of solvents in depolymerization include hydrogen supply [30], promotion of lignin dissolution [31], stabilization of intermediate products, and inhibition of condensation reactions [32]. Researchers have found that a biphasic system of dioxane–water can achieve a liquid yield of 95.6% when using HTaMoO_6_ and Rh/C as composite catalysts for lignin depolymerization. Similarly, when using HPA as a catalyst, all types of heteropolyacid (HPA), Pd/C, H-donor solvents, and reaction temperatures significantly affect the conversion rate of kraft lignin into bio-oil [33]. In addition to external factors, the inherent structural characteristics of lignin also play a crucial role in its transformation.

Lignin depolymerization is a promising area of research for the sustainable utilization of lignocellulosic biomass. However, it is important to consider the sustainability of the reagents, solvents, and catalysts used in the process. For example, some catalysts containing precious metals such as palladium and ruthenium are expensive and scarce, making them less sustainable. In contrast, catalysts containing Earth-abundant metals such as iron and nickel are more sustainable alternatives. Solvents used for lignin depolymerization also need to be considered for sustainability. Traditional organic solvents such as methanol and dioxane can be harmful to human health and the environment, and their production can be energy intensive. Therefore, the use of bio-based and environmentally friendly solvents such as ionic liquids and deep eutectic solvents is gaining attention in the field of lignin depolymerization. In summary, as lignin depolymerization technologies continue to develop, it is crucial to consider the sustainability of the reagents, solvents, and catalysts used. By choosing sustainable alternatives, the lignin valorization process can become more environmentally friendly and economically viable.

Currently, there is a limited number of reviews available that explore the relationship between lignin’s structural features and its depolymerization products. Therefore, this review aims to provide a comprehensive overview of common depolymerization techniques utilized for lignin, as well as the key structural features of lignin, such as the content of β-O-4 linkage and the basic structural units present. Additionally, this review will highlight the impact of C-lignin on the formation of depolymerization products. The information presented herein will be of great value to researchers who seek to design experiments and conduct further research on the mechanisms of lignin depolymerization.

## 2. Lignin Structure and Properties

**Figure 1:**
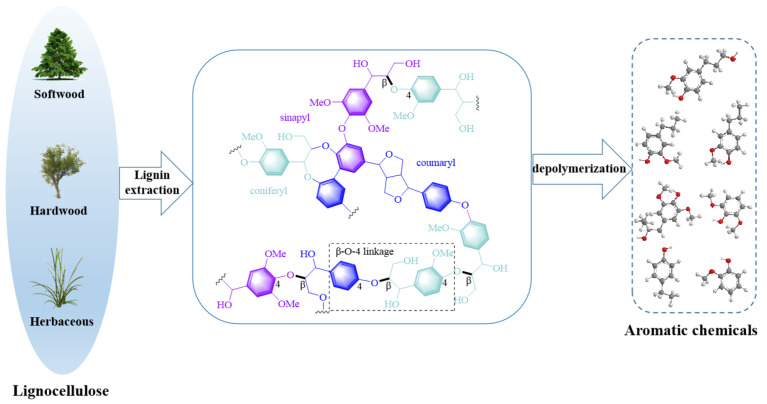
Lignin structure and its transformation.

Lignin is a class of biopolymers that is characterized by its complex, three-dimensional reticulated structure. Its complex formed with carbohydrates, primarily hemicellulose, represents a significant obstacle in the pulping process [34,35]. High temperatures and acid or base catalysts are typically required for lignin extraction. After depolymerization, lignin fragments can be converted into free radicals or ions that can undergo random recombination to form new carbon–carbon bonds [36]. Dilute acid and hot water have also been reported to alter the lignin structure [37]. The lignin extracted by organic solvent methods has high purity, no impurities, and retains most of the β-O-4 structure [38]. Currently, inorganic acids and bases are added in many extraction processes to enhance extraction efficiency. The relative molecular weight of lignin obtained by enzymolysis is the largest, which may be closer to the original structure of lignin [39]. The three fundamental building blocks of lignin, shown in Figure 1, are *p*-hydroxyphenyl, guaiacyl, and syringyl phenylpropane units [40]. The main connection mode between lignin structural units is the C-O bond, followed by the C-C bond [41]. These building blocks are interconnected by various chemical bonds, such as β-O-4, 5-5, β-5, β-1, 4-O-5, and β-β, to form the intricate and complex structure of lignin molecules [42].

Research has demonstrated that the ether bond is the most prevalent linkage among the fundamental unit linkages of lignin, encompassing alkyl aryl ethers, diaryl ethers, and dialkyl ethers [43]. For most natural lignin, the β-O-4 linkage represents the most frequent type of ether bond linkage. However, it is not inherently stable. Under the influence of high temperature and pressure or chemical reagents, β-O-4 ether bonds become susceptible to breakage, resulting in the depolymerization of lignin macromolecules into smaller entities [44]. Therefore, most studies on authentic lignin depolymerization have focused on the β-O-4 structure, and most lignin model compounds have been synthesized based on this structure.

## 3. Study on Lignin Depolymerization

### 3.1. Oxidative Degradation

Oxidative degradation has been shown to have a high depolymerization rate for lignin, and the use of relatively inexpensive oxidants makes it possible to produce single benzene ring compounds containing various functional groups under mild reaction conditions. C-C bond cleavage at different sites in the benzene ring side chain within the oxidation catalytic system results in various products, including phenolic aldehyde, carboxylic acid aromatic, and para-quinone aromatic compounds [45,46,47,48]. Further oxidation can lead to the breakdown of the benzene ring structure, yielding aliphatic carboxylic acid products. The oxidants used in the oxidative degradation process mainly comprise oxygen, ozone, hydrogen peroxide, chlorine dioxide, hypochlorite, metal oxides, and nitrobenzene [49]. The most common catalytic oxidation degradation methods for lignin are electrochemical catalytic oxidation [50], photocatalytic oxidation [51], inorganic salt catalytic oxidation [52], and metal organic catalytic oxidation [16]. Electrochemical catalytic oxidation has recently emerged as a new approach for lignin degradation owing to its low cost, high efficiency, and environmental friendliness [50]. Titanium dioxide is the most frequently employed photocatalyst in photocatalytic oxidation of lignin degradation due to lignin’s unique chemical stability [51]. Inorganic salt catalytic oxidation predominantly relies on the oxidative properties of the catalyst itself, and although the cost is low, the selectivity is restricted, and the yield of depolymerized monomers obtained under this technique is comparatively low [52]. The alkaline oxidation catalysis of lignin for degradation primarily employs dimethyl carbonate as a solvent to esterify lignin’s hydroxyl group, followed by depolymerization with cesium carbonate, resulting in the depolymerization of lignin into small molecule aromatic compounds [53]. However, lignin can be depolymerized into coniferin with a yield of 73.3% by synthesizing titanium oxide photocatalysts, cobalt-based electrocatalysts, and biocatalysts [54]. In addition to conventional oxidants, some blocking agents, such as 2,3-dichloro-5,6-dicyano-1,4-benzoquinone (DDQ), dimethyl sulfoxide (DMSO), and 2,2,6,6-tetramethyl-1-piperidinyloxy (TEMPO), have also been employed as catalysts or oxidants for lignin oxidation depolymerization [55,56]. However, these investigations mainly concentrate on the oxidation depolymerization of lignin model compounds, and the catalytic effect on lignin requires further verification.

Although various methods are currently available for the oxidative depolymerization of lignin, controlling the degree of oxidation remains a significant challenge. Excessive oxidation can result in the formation of low-value products such as carboxylic acids, while vanillin is a product of relatively high value. Furthermore, repolymerization during oxidative depolymerization of lignin is a critical issue that requires further investigation. To address this challenge, the development of effective oxidants or catalysts that can inhibit repolymerization and enable moderate, targeted oxidation of lignin is necessary.

### 3.2. Reductive Degradation

Compared to oxidative methods, the reduction method is more commonly used for catalytic lignin degradation due to its higher yield of degradation products. This is mainly because the degradation products of lignin, when reduced, yield aromatic monomers that are deoxygenated, resulting in simpler products and improved selectivity [57]. The reduction method mainly breaks the C-β-O bond, resulting in typical products such as aromatic derivatives of propylbenzene [58]. Supported noble metal catalysts, such as Pd/C, have been found to be efficient in achieving directed depolymerization of lignin, yielding high yields and selective production of degradation products, such as dihydrosinapyl alcohol and dihydroconiferyl alcohol [59,60]. While transition metals, such as Cu, Fe, and Ni, are cheaper than noble metals and have good catalytic properties, they are commonly used for hydrogenation and reduction depolymerization of lignin. For example, the weakly acidic Ni/SBA-15 catalyst can effectively catalyze the hydrogenolysis of organic solvent lignin, achieving up to 30% bio-oil yield [61]. Ni loaded on an alkaline MgO carrier can also effectively promote the hydrogenolysis of lignin, where a large amount of ether bonds are cleaved to form small molecular products, while the hydrogenation reaction inhibits repolymerization [62]. However, Ni also promotes dehydration and carbonization of the lignin molecule, leading to partial charring of the product. Adding an appropriate proportion of Al_2_O_3_ as a catalyst can significantly improve the yield of single phenols in lignin hydrogenolysis products [29]. Bimetallic catalysts, such as Ni-Au and Zn/Pd/C, have been reported to significantly improve catalytic performance in lignin depolymerization due to their ability to change the electronic and geometric structure of the single metal catalyst surface, affecting its activity, stability, and selectivity [63,64].

There is extensive ongoing research on the use of catalysts for hydrogenation of lignin. However, it is important to note that intermediate products may form during hydrogenation reactions [65,66]. Moreover, ether bond cleavage and hydroxyl group elimination reactions may take place, leading to the formation of methoxyphenolic monomers. Additionally, different catalysts may lead to secondary reactions such as hydrogenation and deoxygenation, resulting in the formation of monophenolic compounds, cyclic alcohols, or cyclic hydrocarbon compounds [67]. It is worth noting that lignin monomer yields obtained through different catalytic methods cannot be easily compared since lignin structure and composition vary depending on the extraction method used and the source plant.

### 3.3. Other Degradation Methods

Various degradation methods have been explored to convert lignin into high value-added chemicals. Oxidative and reductive degradation methods have been shown to produce aldehydes, ketones, acids, and aromatic hydrocarbons, which can undergo further reactions to yield valuable products. Alongside chemical degradation, physical and biodegradation methods have also been investigated for lignin degradation [68,69]. Physical degradation is typically used as a preliminary qualitative treatment to isolate lignin for industrial applications or to obtain raw materials for biomass fuel. However, it is not commonly used for direct lignin degradation. Biodegradation shows promise in both environmental protection and papermaking industries, but it requires careful selection and cultivation of bacteria, which may limit its practical application. Despite these alternative methods, chemical degradation is often favored by researchers due to its ability to degrade lignin at the molecular level. Nevertheless, there are significant challenges to the industrial utilization of lignin by chemical degradation, such as high energy consumption, propensity for reaggregation, and difficulties with separation. Therefore, the high-value utilization of lignin remains an important research topic in environmental protection. Future research should aim to improve lignin degradation systems and the application of its degradation products.

## 4. Structural Characteristics–Reactivity Relationships in Monomer Yield

### 4.1. Effect of Lignin β-O-4 Structure on Monomer Yield

**Figure 2:**
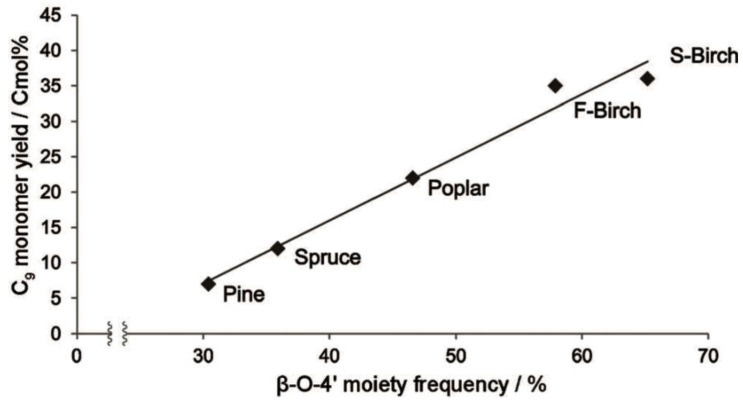
The dependency of monomer yield on β-O-4 content in Pd/C catalysis [70].

Pretreatment and separation methods for lignin often lead to significant changes in its structure. The main linkage bond between the structural units of lignin is the β-O-4 structure, which comprises about 50% in coniferous wood and 50–70% in broad-leaved wood [36]. The β-O-4 content in lignin structural units is influenced by plant species, growth site, growth cycle, and growth environment. C_β_-O bond breaking is the primary method for catalytic degradation of lignin, and the highest theoretical monomer yield for lignin degradation depends on the degradable β-O-4 content, which is usually the square of the β-O-4 content. The biomass feedstock itself can impact lignin degradation. Samec studied the reductive degradation of various fast-growing wood feedstock under Pd/C catalyst, ethanol/water solvent, and hydrogen-free conditions, and found that the yield of lignin-degraded phenol monomer was linearly related to the β-O-4 content. Coniferous pine lignin, with less β-O-4 linkage, resulted in only 7% phenol monomer, while broad-leaved wood lignin, with more β-O-4 linkage, produced a higher yield of phenol monomer. For example, the β-O-4 content of birch (S-Birch) from Sweden reached 65%, resulting in a phenol monomer yield of 36% [70]. As shown in Figure 2, the β-O-4 content has a significant impact on the yield of phenol monomer during lignin degradation.

**Figure 3:**
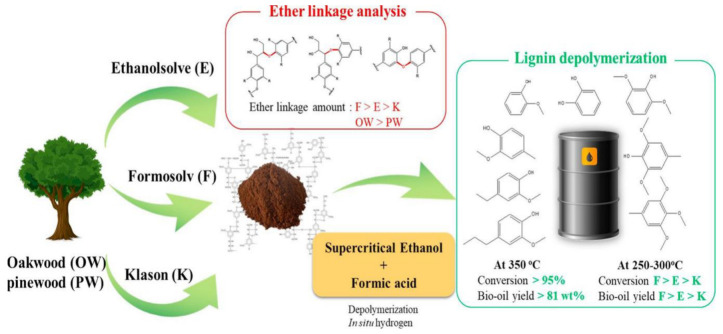
Lignin with different ether bond content was depolymerized into oily products [71].

Lignin raw materials display differences in their ether bond content. As shown in Figure 3, six lignin samples were produced from oak (OW, hardwood) and pine (PW, softwood) using three different delignification techniques (ethanol organic solvent, formasolv, and Klason lignin separation method). The ether bond content in OW-derived lignin was found to be about three times higher than in PW-derived lignin due to the presence of mustard alcohol units in the former. The order of ether bond content in lignin isolated by different methods was formasolv > ethanol organic solvent > Klason. Lignin samples were depolymerized by a mixture of supercritical ethanol (scEtOH) and formic acid (HCOOH) at temperatures ranging from 250 to 350 °C. Regardless of the lignin type, high conversions (>95%) and high bio-oil yields (>81 wt%) were achieved at 350 °C, indicating that the combined use of scEtOH-HCOOH was very effective in depolymerizing various types of lignin. At lower temperatures of 250–300 °C, the degree of lignin conversion and bio-oil yield were highly dependent on the number of ether bonds present [71].

**Figure 4:**
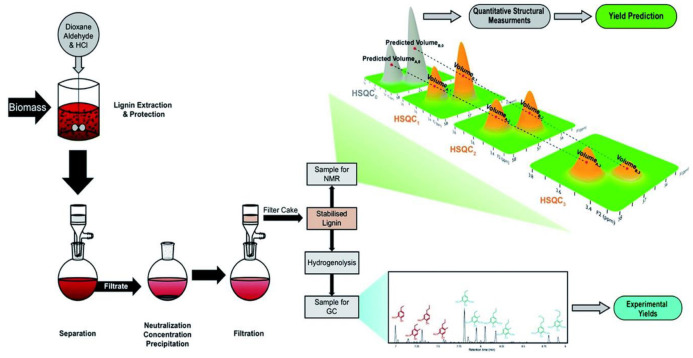
Catalytic conversion of lignin and its characterization [73].

**Figure 5:**
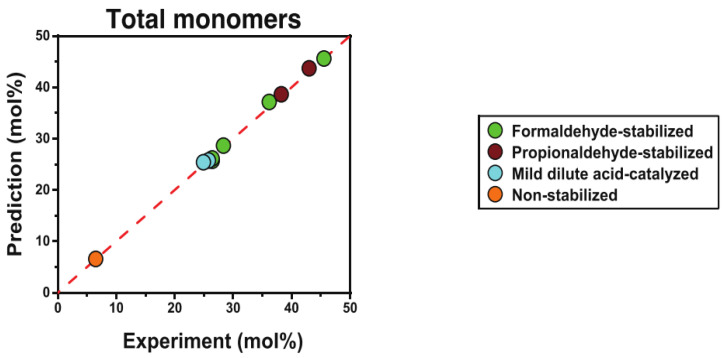
Comparison of predicted and experimental yields of lignin depolymerization under different pretreatments [73].

The content of ether bonds in lignin can have a significant impact on its catalytic conversion. In a study, lignin with varying alkyl-aryl ether bond contents was depolymerized using alumina-supported platinum catalyst. The results indicated that the ratio of β-O-4 bonds had a crucial influence on both the yield and the properties of the monomer products. Highly condensed lignin mainly produced non-alkylated phenolic products, while uncondensed lignin mainly produced phenolic products that retained three-carbon side chains. The yields of these phenolic products retaining the three-carbon side chains were significantly lower than the maximum potential yields calculated by selective cleavage of the alkyl-aryl ether bonds using the thioacid method, suggesting that there is still scope for improvement in yield. Although the catalytic conversion rate increased with increasing ether bond content in the lignin structure, optimizing the catalytic depolymerization process is still necessary to reduce side reactions [72]. To further investigate the relationship between ether content and monomer yield, a series of lignin samples with different ether contents (6–46%) were prepared, and a modified HSQC-NMR method was used to determine the total number of β-O-4 linkage bonds in the samples (as shown in Figure 4). The final depolymerization yield was accurately predicted (error < 4%) using a simple ether cleavage model. This finding demonstrated a direct causal relationship between ether content and monomer yield. Although the theoretical monomer yield of lignin based on ether bond breaking can usually predict the monomer yield of natural lignin, the large chain length of natural lignin and the random distribution of ether and C-C bonds mean that further experimental optimization is needed, as some of the data presented in Figure 5 indicate [73].

**Figure 6:**
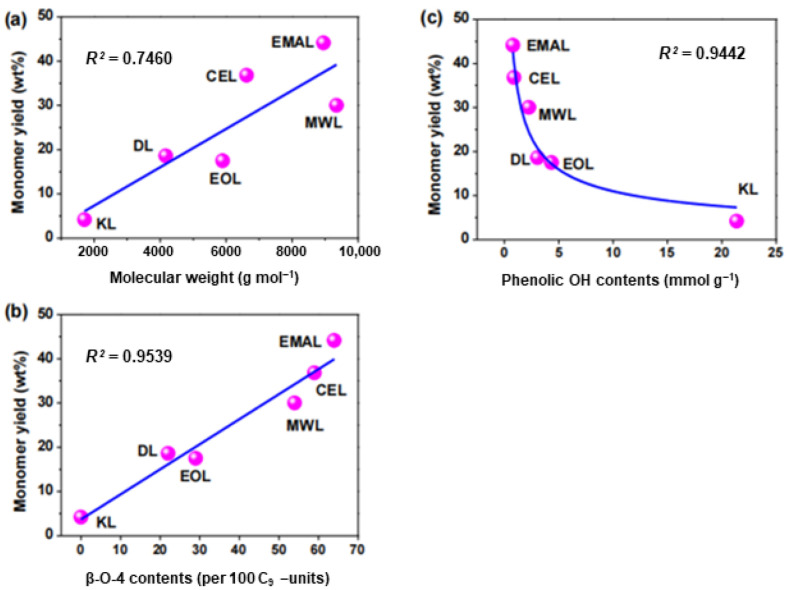
The correlations among the quantified aromatic monomer yields and the lignin structural features. (**a**) Linear correlation between lignin monomer yield and molecular weight of 6 lignin samples; (**b**) Linear correlation between lignin monomer yield and β-O-4 content in 6 lignin samples; (**c**) Correlation between lignin monomer yield and phenolic hydroxyl content in 6 lignin samples [74].

A recent study has shown that, in addition to the correlation between ether bond content and catalytic conversion, other structures of lignin are also affected to varying degrees [74]. In this study, six lignin samples were prepared from eucalyptus, including three natural lignin samples (EMAL, enzymatic mild acidolysis lignin; CEL, cellulolytic enzyme lignin; and MWL, milled wood lignin) and three industrial lignin samples (EOL, ethanol organosolv lignin; DL, dioxane acidolysis lignin; and KL, kraft lignin). Monomeric products were obtained via catalytic hydrogenolysis reactions. The results indicated that the catalytic hydrogenolysis reaction of EMAL was consistent with previous reports, with the main products being 4-n-propanol guaiacol (9.6 wt%) and 4-n-propanol eugenol (28.1 wt%), which together accounted for 76% of the total monomers and exhibited high monomer selectivity. The theoretical maximum yields of the aromatic monomer products were evaluated using 2D HSQC NMR results, and the monomer yields of the three industrial lignins were slightly higher than the theoretical yields, probably because the theoretical yield calculation method is more applicable to natural lignin with more abundant β-O-4 bonds. The correlation between the quantified aromatic monomer yields and the structural characteristics of lignin was further investigated, and it was found that the correlation values of R^2^ between β-O-4 content and phenolic hydroxyl content were greater than 0.94 (as shown in Figure 6b,c), but the correlation values of R^2^ between monomer yields and molecular weights were slightly lower than 0.75 (as shown in Figure 6a). The results show that natural lignin samples have a higher monomer yield due to the abundant β-O-4 bond, while industrial lignin samples require higher energy to obtain a higher monomer yield due to the partial/total fracture of ether bond during the separation process. In addition, a negative correlation was found between lignin monomer yield and phenolic hydroxyl group.

The compound vanillin is highly valued and widely used as a spice in the food and cosmetics industries [75]. In a recent study, three main industrial lignin and two enzymatic lignin samples were oxidized by nitrobenzene in an alkaline solution, and the highest vanillin yields were obtained from the enzymatic lignin (13.39%) and industrial lignin (11.64%). Analysis results indicated that the β-O-4 bond in lignin greatly influenced the corresponding aldehyde yield. Lignin model compounds with the β-O-4 bond as the main connecting bond were also oxidized by nitrobenzene, and over 40% vanillin yield was obtained from these model compounds. This finding suggests that lignin with a high β-O-4 bond content can achieve a higher vanillin yield, providing guidance for the selective degradation of lignin into aromatic chemicals [76].

### 4.2. Effect of Other Structures of Lignin on Monomer Yield

The yield of monomeric compounds obtained by lignin depolymerization is not only related to the β-O-4 linkage bond content between lignin structural units, but also to the basic lignin structural units. Several studies have suggested that the yield of monophenolic compounds obtained from lignin depolymerization is correlated with the content of S and G types in lignin. Lignin monomers with more S-type structural units are easier to depolymerize, resulting in a higher yield of monophenolic compounds.

**Figure 7:**
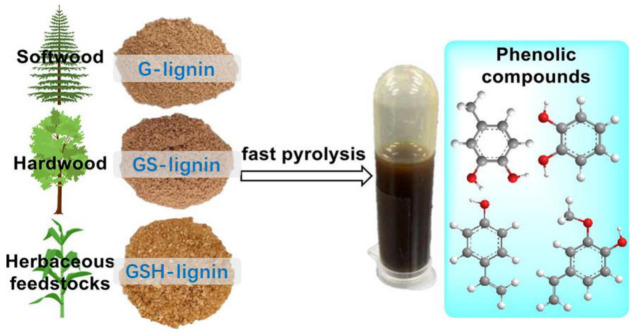
Lignin from different sources is rapidly pyrolyzed into phenolic compounds [77].

In Figure 7, the study on the structure–activity relationship between lignin and its pyrolysis products is presented. Mild acid hydrolyzed lignin (MAL) isolated from various raw materials, such as softwoods (pine), hardwood (eucalyptus), and herbaceous materials (corn straw and bamboo), was characterized. The detailed characterization of the lignin structure revealed that eucalyptus MAL contained guaiacyl (G) and syringyl (S) units, whereas pine MAL was rich in G units. Additionally, corn straw and bamboo MAL, apart from G, S, and p-hydroxyphenyl (H) units, also consisted of trioctanoic and hydroxycinnamic acids (ferulic and p-coumaric). The lignin samples from these plants carried p-coumarate groups in the lignin side chain at Cγ. The results of the rapid pyrolysis of different lignin sources showed a wide range of aromatic compounds due to the selective breaking of lignin bonds. Notably, herbaceous MAL provided higher amounts of phenolic compounds, with 4-vinyl phenol being the main pyrolysis product, indicating an efficient cleavage of the C-O bond coupled with decarboxylation reactions in lignin. Understanding the structure–activity relationship between lignin and its pyrolysis products is essential for the thermochemical conversion of lignin, and this study will guide the rational design of thermal chemical processes to enhance the production of phenolic compounds from lignin via rapid pyrolysis [77].

To investigate the degradation and potential use of lignin’s basic structural units, the chemical structures of poplar, pine, and rice straw samples were analyzed, and the possibility of producing phenolic monomers using phosphotungstic acid (PTA) catalyst was explored. The study revealed that lignin components with different ratios of H, G, and S had varying structures. Among the lignin samples extracted from the three sources, poplar had the highest molecular weight and β-O-4 aryl ether content, followed by pine and rice straw. Upon depolymerization using PTA catalyst, the yields of phenolic monomers were 8.06 wt% (poplar), 5.44 wt% (pine), and 4.52 wt% (rice straw). Furthermore, the H/G/S ratios in the phenol monomers differed, indicating that the S, G, and H structural units underwent continuous interconversion during the reaction. These findings shed light on the transformation of lignin’s basic structural units during degradation and have potential implications for the production of phenolic monomers via thermochemical processes [78].

The relationship between the conformational features of lignin and its degradation products has been investigated using complex lignin structures and a broad range of oxidative degradation products. The multivariate linear estimation (MVLE) method was applied to simulate the reaction products of seven lignin samples, including monophenolic compounds (MPCs) and dibasic acids (DCAs), revealing the quantitative relationship between the yield of monophenolic compounds obtained from the reaction and four structural parameters: methoxyl content, aliphatic hydroxyl content, ether bond content, and molecular weight during the oxidative depolymerization of lignin. The team discovered that higher methoxyl content hinders MPC formation, while aliphatic hydroxyl content has a similar effect, whereas ether bonding promotes MPC formation, and lignin molecular weight has a natural logarithmic relationship with the monophenolic products. Among the six lignin samples, methoxyl content had a negligible effect on DCA yield, and molecular weight had a similar impact, but an increase in ether bonding was linked to an increase in monophenolic products. The MVLE model’s results confirm that lignin oxidative depolymerization is influenced by a set of structural variables, rather than individual factors, which provides a valuable new tool for studying the effect of lignin structure on MPC products. However, the application of this method to DCA formation is more complex and requires further exploration by researchers [79].

**Figure 8:**
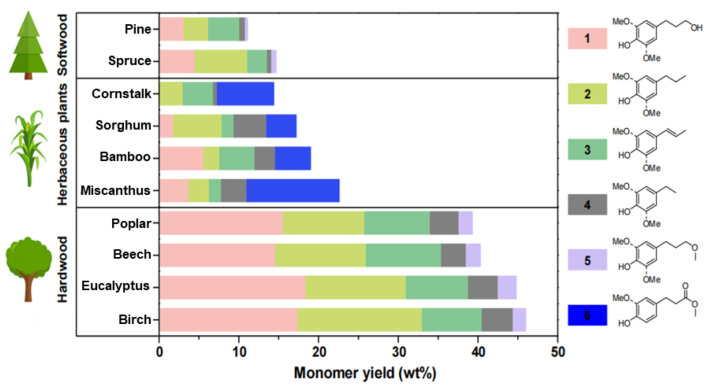
Effects of lignin from different sources on catalytic hydrolysis of lignocellulosic biomass [80].

Additionally, it should be noted that the source of lignin also plays a significant role in determining the yield of phenolic monomers. In a recent study, phenolic monomers were produced from various lignocellulosic materials using a carbon nanotube-supported ruthenium (Ru/CNT) catalyst, which exhibited high activity, reusability, and compatibility with different biomass feedstocks. The results, as depicted in Figure 8, showed that the yield of aromatic monomers is closely related to the source of lignin, with the highest yield obtained from hardwood, followed by herbaceous plants and softwood [80].

In summary, lignin structures can exert varying degrees of influence on both monomer yield after lignin degradation and selectivity for specific monomers. Researchers have developed more reasonable simulation predictions in their respective fields, including systematic data processing before prediction, which facilitates in-depth investigation of the entire lignin degradation process. However, the complex chemical structure of lignin, different methods of separation and extraction, and different degradation techniques present challenges and are key factors in realizing the high value of lignin utilization.

### 4.3. Depolymerization of C-Lignin

**Figure 9:**
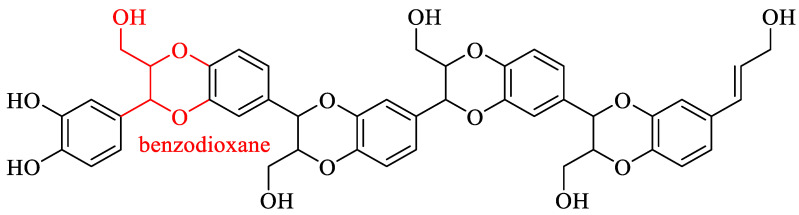
Structure of C-lignin [83].

The biomolecule catechyl lignin (C-lignin) is composed of a single caffeol structural unit linked by benzodioxane and exhibits linearity, homogeneity, and strong acid resistance [81]. The structure of C-lignin is illustrated in Figure 9. Currently, C-lignin has been identified primarily in the seed coats of certain plant species belonging to the families Orchidaceae, Cactaceae, Euphorbiaceae, and Drametaceae [82,83].

**Figure 10:**
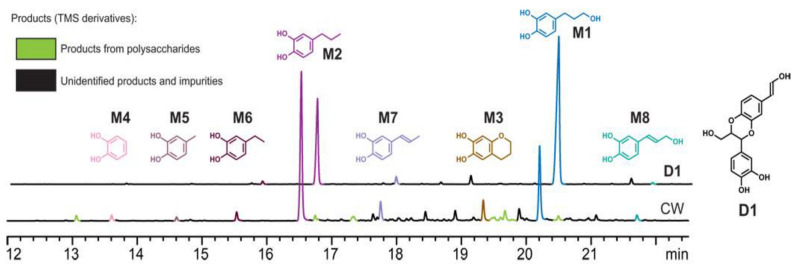
Dimeric compounds D1 and CW hydrolysis products [84].

**Figure 11:**
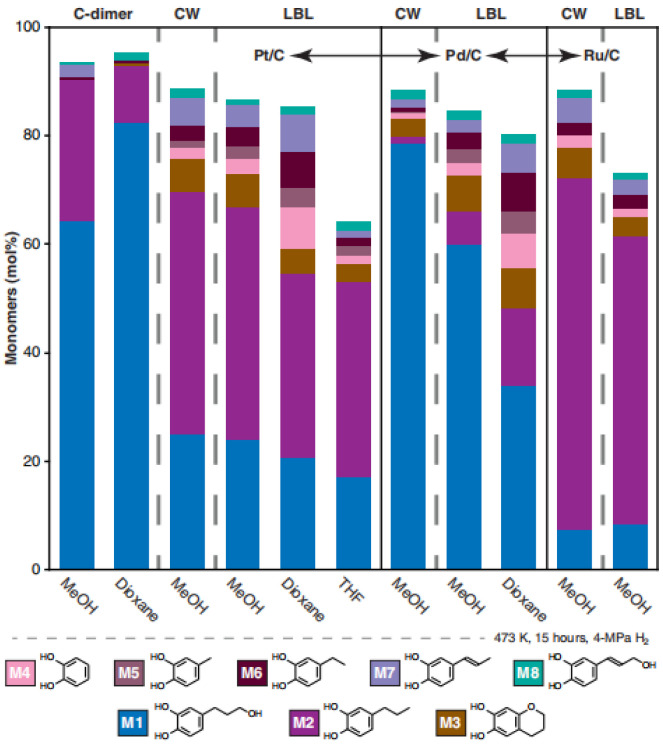
Hydrogenolysis monomers from different catalyst and solvent combinations [84].

Li was the first to report the hydrolysis products of vanilla seed coat (CW) and its dimer model (D1), and the main products, catechol monomers M1 and M2, were identified by comparison with authentic synthetic standards. The minor product, M3, was the cyclization product of M1, as shown in Figure 10 and Figure 11. Catalyst and solvent choice were compared, and it was concluded that the highest monomer yield and reaction selectivity were achieved using Pd/C or Ru/C catalysts with methanol as the solvent. The selectivity of catechol monomer M1 prepared by Pd/C was 89%, and the selectivity of catechol monomer M2 prepared by Ru/C was 74%. Increasing the hydrolysis reaction time from 3 h to 15 h increased the lignin conversion and monomer yield by about 10% [84].

More efficient separation and selective degradation of C-lignin can be achieved by using different pretreatment methods to separate C-lignin and G/S-lignin from various sources, such as Jatropha curcas, stone chestnut, oleander, and castor seed coats. The authors concluded that dioxane and dilute hydrochloric acid extraction was the optimal solution for the splitting of C-lignin and G/S-lignin, based on a comparison of separation efficiency, operational steps, and energy consumption. This method was able to almost completely separate the two lignins in Jatropha curcas seeds, and was extended to other plants in the family Euphorbiaceae that contain both C-lignin and G/S-lignin. The isolated C-lignin was then catalytically hydrogenated by Pd/C to yield a single catechol product with up to 97% selectivity. This approach showed superior catalytic activity and selectivity when compared to feedstocks containing both C-lignin and G/S-lignin [85].

**Figure 12:**
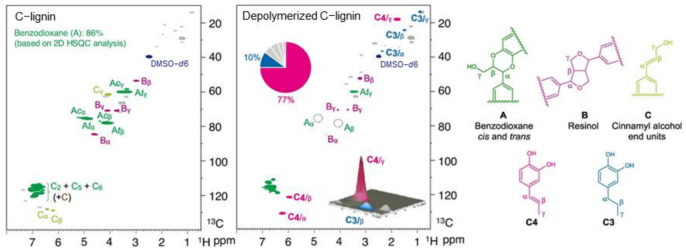
The 2D NMR spectrum of C-lignin and oily product [86].

In addition to Pd/C catalysts, catalysts containing Ru metal are another type of catalyst that can be used for lignin degradation. The addition of 0.2% Ru can reduce the C=C hydrogenation capacity and the catalyst cost. These catalysts can efficiently catalyze the hydrogenolysis of C-lignin by cleaving the carbon–oxygen bond in the benzodioxane ring, resulting in a high yield of catechol. Among them, propylene-catechol has a unique selectivity of up to 77%, and its NMR spectrum is shown in Figure 12. The catalyst also has good reusability in lignin depolymerization, as it can be recycled at least six times. Detailed studies of the model compound suggest that the caffeic alcohol produced by the simultaneous cleavage of the carbon–oxygen bond of the benzodioxane ring might be an intermediate in the C-lignin hydrogenation reaction. These experiments demonstrate that atomically dispersed catalysts can catalyze not only small molecule reactions but also drive the conversion of abundant renewable biopolymers [86].

## 5. Conclusions

Based on the complexity of lignin’s structure, the effects of various pretreatment methods on the lignin structure have limited the lignin depolymerization methods and hindered efficient utilization of lignin. Therefore, it is essential to conduct in-depth research on the lignin structure while focusing on yield and selectivity. Comparing and analyzing the theoretical values estimated by researchers with real experimental values is critical to record the advantages and disadvantages of different systems for data processing. When drawing on researchers’ prediction methods, we should summarize their prediction methods and organize them in terms of lignin pretreatment and degradation methods according to raw materials, including catalysts and solvents used. Doing so can systematically combine these methods with researchers’ prediction methods and be used to conduct corresponding experiments. To explore the influence of different lignin structures on the degradation process and degradation products, appropriate model compounds should be synthesized, and the appropriate prediction methods should be selected to analyze the different influencing factors. It is crucial to understand the limitations of these methods and means of future improvements to achieve high-value utilization of lignin.

## Data Availability

Not applicable.
